# Attitude toward patients with mental disorders: what is going on amongst Iranian pharmacies?

**DOI:** 10.1186/s40359-024-01604-0

**Published:** 2024-03-06

**Authors:** Fatemeh Shirvaniyan, Negin Eissazade, Seved Vahid Shariat, Noushin Khademoreza, Masoomeh Daneshvar, Mohammadreza Shalbafan

**Affiliations:** 1 Mental Health Research Center, Psychosocial Health Research Institute, Department of Psychiatry, School of Medicine, Iran University of Medical Sciences, Tehran, Iran; 2 Brain and Cognition Clinic, Institute for Cognitive Sciences Studies, Tehran, Iran; 3 Mental Health Research Center, School of Behavioral Sciences and Mental Health (Tehran Institute of Psychiatry), Iran University of Medical Sciences, Tehran, Iran

**Keywords:** Stigma, Mental disorder, Pharmacists, Pharmacy, Pharmacy technician

## Abstract

**Introduction:**

As the incidence of mental disorders continues to rise, and pharmacy staff can significantly affect the willingness of patients with mental disorders to seek help; we aimed to evaluate the stigmatizing attitude of the pharmacy staff toward these patients in Iran.

**Methods:**

We conducted this cross-sectional study between April 2020 and December 2021 in Tehran, Iran, and included pharmacists, pharmacy technicians and pharmacy students, with the experience of working in a pharmacy for at least three months. The social distance scale (SDS) and dangerousness scale (DS) were used to measure the stigmatizing attitude of the participants. Higher scores indicated more stigmatizing attitudes.

**Results:**

We included a total of 186 participants with a mean age of 32.97 ± 9.41 years, of which 101 (54.3%) were male, and 75 (40.3%) were pharmacists, 101 (54.3%) were pharmacy technicians, and 8 (4.3%) were pharmacy students. The mean SDS score was 14.2 ± 4.13, and the mean DS score was 33.85 ± 8.92. The greatest tendency for social distance was reported for a patient with a mental disorder, ‘*being the caretaker of their children for an hour or two’* and ‘*marrying their children.’* The most perceived dangerousness was reported for a patient with a mental disorder *‘owning a gun.’* Positive personal history of psychopharmacological treatment was statistically correlated with lower DS (P = 0.001) and SDS (P = 0.007) scores. Positive family history of psychiatric inpatient admission was significantly correlated with higher DS (P = 0.05) and SDS (P = 0.03) scores. Higher rates of ‘received psychiatric prescriptions per month’ was associated with lower DS scores (P = 0.04).

**Conclusion:**

Our participants did not have an overall positive attitude toward patients with mental disorders. Although, compared to previous studies, they held a more positive attitude. Positive personal history of psychopharmacological treatment predicted a more positive attitude and positive family history of psychiatric inpatient admission predicted a more negative attitude.

## Background

More than 450 million individuals worldwide are affected by mental disorders, with less than two-thirds seeking treatment or care. Untreated mental disorders may worsen and lead to impaired daily function, decreased quality of life, and even suicide. Stigma toward patients with mental disorders has long been a barrier to seeking mental healthcare. Stigma stems from misconception and prejudice, leading to social exclusion, further exacerbating reluctance to seek care [1–3]. Stigmatization can simultaneously occur on intrapersonal, interpersonal, and structural levels [4]. It affects causation, coping styles, treatment seeking attitude, trust, overall health status, communication with the psychiatrist, and primary care [5].

Stigma reduction strategies vary by specific societal factors, e.g., culture, religion, politics, and socio-economics [6–8], as they shape the public attitude and the patients’ willingness to seek help [9]. Iran is a multicultural Middle Eastern Islamic country with a population estimated at approximately 89.5 million, and diverse ethnic communities. The prevalence of mental disorders in Iran has been reported as 31.03% [10, 11]. Moreover, the stigma caused by mental disorder in Iran is as high as 40% [12].

Stigma also affects healthcare settings, more specifically, pharmacies. As a result, a negative provider-patient relationship will be formed and patients may be discouraged from seeking help [13]. The limited research regarding the attitude of pharmacy staff toward patients with mental disorders has shown an overall negative attitude [14–21]. As reported by previous studies, the contributing factors include the healthcare workers’ negative attitude and behavior, lack of awareness, therapeutic pessimism and lack of skills. Additionally, stigma reduction is not integrated into the training curriculum of healthcare workers [22, 23]. However, in a pharmacy setting, practicing pharmacists can be vigilant for early detection of mental disorders, signs of medication misuse, and inappropriate prescriptions by physicians. Moreover, they can be a resource of information for patients regarding the appropriate use of medications, and also, receive feedback for improving medication adherence [13, 14].

As the stigma surrounding mental health in Iran persists as a barrier of mental healthcare, due to cultural, religious, and social factors [8, 24], and the pharmacy staff significantly affect the willingness of patients to seek care, we aimed to evaluate the stigmatizing attitude of pharmacy staff toward patients with mental disorders in Iran.

## Methods

### Design and participants

We conducted this cross-sectional study between April 2020 and December 2021. We included 186 participants from pharmacists, pharmacy technicians and pharmacy students in Tehran, Iran, (as Tehran is a multicultural city, its population can be representative of the Iranian population) who had worked in a pharmacy for at least three months.

### Data collection

Demographic data were collected. The Persian version of the Social Distance Scale (SDS) and Dangerousness Scale (DS) were used to measure the stigmatizing attitude of participants.

### Instruments

SDS consists of seven questions that evaluate the tendency to interact with a patient with a mental disorder. It is scored using a 4-point Likert scale, ranging from 0 to 21. Higher scores indicate a greater tendency for social distance. The validity and reliability of the Persian version of this questionnaire have been reported as follows: Cronbach’s alpha coefficient of 0.92, test-retest reliability coefficient of 0.89, and content validity coefficient of 0.75 [24–26].

DS consists of eight questions that evaluate the participant’s perception of the dangerousness of a patient with a mental disorder. It is scored using a 7-point Likert scale, ranging from 7 to 56. Higher scores indicate greater levels of perceived dangerousness. The validity and reliability of the Persian version of this questionnaire have been reported as follows: Cronbach’s alpha coefficient of 0.96, test-retest reliability coefficient of 0.88, and content validity coefficients of 0.77 [24–26].

### Data analysis

Regarding an SDS mean score of 19.65 ± 3.97, type I error of 0.05, and a 10% attrition bias, a sample size of 186 was calculated.

Data are descriptively reported as frequency (percentage) and mean ± SD. The Pearson and Spearman coefficients were used to assess associations. Additionally, Point-biserial correlation was used to determine the relationship between dichotomous variables and DS and SDS scores. One-way ANOVA was used to assess the relationship between categorical variables and DS and SDS scores. All data were analyzed using SPSS (version 25, SPSS Inc., Chicago, IL, USA). A P-value equal to or less than 0.05 was considered statistically significant.

## Results

We included 186 participants, including 75 (40.3%) pharmacists, 101 (54.3%) pharmacy technicians, and 8 (4.3%) pharmacy students, with a mean age of 32.97 ± 9.41 years (range: 18–63 years), of which 101 (54.3%) were male.

Regarding the past psychiatric history of the participants, 24 (12.9%) had a positive personal history of psychopharmacological treatment, 2 (1.1%) had a positive personal history of psychiatric inpatient admission, and 43 (23.1%) had a positive family history of psychopharmacological treatment and 23 (12.4%) had a positive family history of psychiatric inpatient admission. Baseline data are presented in Table 1.

**Table 1:** Baseline data of the participants

Variables		Frequency (Percentage)
Gender	Female	85 (45.7%)
	Male	101 (54.3%)
Education	Undergraduate	2 (1.1%)
	Diploma	12 (6.5%)
	Associate’s degree	17 (9.1%)
	Bachelor’s degree	58 (31.2%)
	Master’s degree	17 (9.1%)
	Doctorate degree	80 (43%)
Work experience at pharmacy	3–12 months	39 (21%)
	1–5 years	75 (40.3%)
	5–10 years	33 (17.7%)
	More than 10 years	39 (21%)
Personal history of psychopharmacological treatment	Negative	162 (87.1%)
	Positive	24 (12.9%)
Family history of psychopharmacological treatment	Negative	143 (76.9%)
	Positive	43 (23.1%)
Personal history of psychiatric inpatient admission	Negative	184 (98.9%)
	Positive	2 (1.1%)
Family history of psychiatric inpatient admission	Negative	163 (87.6%)
	Positive	23 (12.4%)
Role at pharmacy	Pharmacists	75 (40.3%)
	Pharmacy technician	101 (54.3%)
	Pharmacy student	8 (4.3%)
	Others	2 (1.1%)
Received psychiatric prescriptions per month	Less than 10%	28 (15.1%)
	10–30%	62 (33.3%)
	30–60%	49 (26.3%)
	60–90%	26 (14%)
	More than 90%	21 (11.3%)
Accessible mental healthcare facility (within 1 km)	Psychiatric hospital	51 (27.4%)
	Psychiatric clinic	34 (18.3%)
	Uncertain	49 (26.3%)
	None	52 (28%)

The mean DS score was 33.85 ± 8.92, within a range of 9 to 54. The mean SDS score was 14.2 ± 4.13, ranging from 5 to 21. Higher DS scores were significantly correlated with higher SDS scores (Pearson’s *r* = 0.671, *P* < 0.001).

The greatest tendency for social distance was reported for a patient with a mental disorder *’being the caretaker of their children for an hour or two’* (*N* = 129, 69.35%) and *’marrying their children’* (*N* = 122, 65.59%). The most perceived dangerousness was reported for a patient with a mental disorder *‘owning a gun’* (*N* = 64, 34.4%). Positive personal history of psychopharmacological treatment was statistically correlated with lower DS (*P* = 0.001) and SDS (*P* = 0.007) scores. Positive family history of psychiatric inpatient admission was significantly correlated with higher DS (*P* = 0.05) and SDS (*P* = 0.03) scores. Higher rates of ‘received psychiatric prescriptions per month’ was associated with lower DS scores (*P* = 0.04). No other significant correlation was found (Table 2).

**Table 2:** Correlations between baseline data and DS and SDS scores of the participants

Variables	DS scores		SDS scores	
	Correlation coefficient	P-value	Correlation coefficient	P-value
Gender	-0.006^a^	0.93	0.034^a^	0.64
Age	-0.069^a^	0.34	0.085^a^	0.24
Education	-0.038^b^	0.6	− 0.0.056^b^	0.44
Work experience at pharmacy	-0.092^a^	0.21	0.026^a^	0.72
Personal history of psychopharmacological treatment	-0.232^a^	0.001*	-0.197^a^	0.007*
Family history of psychopharmacological treatment	0.035^a^	0.63	0.094^a^	0.20
Personal history of psychiatric inpatient admission	-0.069^a^	0.35	-0.043^a^	0.55
Family history of psychiatric inpatient admission	0.144^a^	0.05*	0.160^a^	0.03*
Received psychiatric prescriptions per month	-0.147^b^	0.04*	-0.044^b^	0.54
Accessible mental healthcare facility (within 1 km)	0.103^a^	0.22	0.129^a^	0.13

^a^Pearson’s r

^b^Spearman’s rho

*Significant correlations with p-values less than 0.05

Role at pharmacy had no significant correlation with neither DS scores (*P* = 0.93) nor SDS scores (*P* = 0.65).

## Discussion

We aimed to assess the stigmatizing attitude of pharmacy staff toward patients with mental disorders and its possible correlation with their demographics. Positive personal history of psychopharmacological treatment and higher rates of ‘received psychiatric prescriptions per month’ predicted a more positive attitude. Positive family history of psychiatric inpatient admission predicted a more negative attitude.

Our pharmacy staff did not have an overall positive attitude toward patients with mental disorders. Although, compared to previous studies, they held a more positive attitude. Our participants had a mean SDS score of 14.2 ± 4.13. However, O’Reilly et al. (2015) studied a group of pharmacists in Australia and reported a higher mean SDS score (17.81 ± 3.79) [16]. In addition, Bell et al. (2010) studied pharmacy students across different countries. They reported negative attitudes, with mean SDS scores of 19.65 ± 3.97 in Australia, 19.61 ± 2.92 in Belgium, 18.75 ± 3.57 in India, 18.05 ± 3.12 in Finland, and 20.90 ± 4.04 in Estonia and Latvia [17]. Moreover, Liekens et al. (2010) reported that community pharmacists in Belgium stigmatize patients with depression [18]. In contrast to our study, Rimal et al. (2022) and Phokeo et al. (2004) studied groups of pharmacists in New Zealand and Canada, respectively, and reported that most participants showed positive attitudes toward mental disorders [19, 20]. Middle Eastern countries have historically been known for their heightened stigmatizing attitudes toward mental disorders due to cultural, religious, and societal factors. However, in recent years, there has been a significant shift toward improvement through awareness campaigns and increased access to mental health education. Additionally, the global discourse on mental health has led to more open conversations and the recognition of the importance of mental health [8–10, 24].

We did not find any significant associations between age and gender, and stigmatizing attitude. However, O’Reilly et al. (2015) reported that male pharmacists were more willing to get involved with these patients [16], and Liekens et al. (2011) reported that older age was a predictor of more negative attitudes [18]. Moreover, we did not observe any significant associations between education level and stigmatizing attitudes. However, previous studies have reported that individuals with higher education levels tend to exhibit a more positive attitude toward patients with mental disorders [26]. These differences may be due to having a small sample size and societal and cultural differences.

Positive family history of psychiatric inpatient admission was significantly correlated with increased stigma. Similarly, Shahveisi et al. (2007) have reported that the intensity of stigma increases withthe history of more than onehospitalization [27]. Moreover, Phillips et al. (2002) have reported that a moderate-to-severe stigmatizing attitude was observed in 26% of the families with patients with mental disorders in their study [28]. A family member with a mental disorder may impact others’ time, energy, emotions, finances and daily activities, leading to evoked strong fears [29]. Facing the relapse or exacerbation phase of patients with mental disorders can cause stigma within families, as the patient may manifest aggressive behavior. However, it has previously been reported that poor family dynamics can lead to increased stigma, potentially contributing to decreased quality of life of the affected individual [30]. Encouraging positive interactions and open discussions about mental disorders within families can help create a supportive and understanding environment.

We found that higher rates of ‘received psychiatric prescriptions per month’ was associated with lower DS scores, indicating a less stigmatizing attitude. This finding supports previous studies that have stated personal contact with patients with mental disorders can lead to decreased stigma [31, 32]. However, contact with patients with mental disorders should be consistent and interactive for preconceived stereotypes to be challenged [33]. Moreover, in line with our study, Giannetti et al. (2018) reported that they observed the willingness to provide services to patients with mental disorders among pharmacists. However, decreased comfort/confidence was reported as a barrier [21]. A potential contributing factor is that pharmacists probably interact with these patients in their remission phase or interact with patients with favorable medication adherence, leading to a more positive attitude among them.

We found that positive personal history of psychopharmacological treatment was statistically correlated with reduced stigma. Similarly, previous studies have reported that pharmacists with personal experience with mental disorders show a more positive attitude [21]. Personal experience of psychopharmacological treatment can lead to developing empathy and reduced misconceptions. Additionally, pharmacists with personal history of psychopharmacological treatment know that the medications can efficiently help control the symptoms.

Similar to the study of O’Reilly et al. (2015), we found that the greatest tendency for social distance was reported for a patient with a mental disorder *‘being the caretaker of their children for an hour or two’* and *‘marrying their children’* [16]. These results offer an intriguing contrast, considering that instances of child abusepredominantly involve family members and caretakers [34]. We also found that the most perceived dangerousness was reported for a patient with a mental disorder *‘owning a gun’.* However, it is of note that no substantial record exists for gun-related homicides involving patients with severe mental disorders over the past decades in Iran. Also, private gun ownership is prohibited in Iran [35]. Mass media might have enhanced this inaccurate belief. Moreover, Bell et al. (2010) reported that the perception of *‘unpredictability’* in Australia, *‘they will never recover’* in India, *‘dangerousness’* in Finland were primarily associated with stigmatizing attitude. However, *‘unpredictability’* was associated with lower SDS scores in Belgium [17]. These findings can be justified in the context of cultural and religious differences.

## Limitations

We had a small sample size. Geographical sampling bias may have limited the generalizability of the findings, as beliefs and attitudes can vary across regions. Also, we had a restricted sample with an admission history, which also limited the generalizability of the findings. Additionally, we employed self-reported questionnaires, which could have been affected by response bias. Future research with large sample sizes across different regions of Iran should be conducted to enhance the reliability and applicability of our findings.

## Implications for research and policies

Research has shown that educational courses and involvement with patients can help improve the attitudes of pharmacists and pharmacy students toward patients with mental disorders and encourage patients to seek help [36, 37]. Only a few studies have used reliable methods to assess pharmacists’ training needs regarding mental disorders and treatment options [36–40]. Further studies are needed to help figure out the exact necessary components of the curriculum. Also, it is suggested to conduct interventional studies to evaluate anti-stigma strategies encompassing education (e.g., campaigns, courses and workshops), personal contact programs, media engagement, advocacy efforts and policy changes that enhance mental health support and accessibility.

## Conclusions

The pharmacy staff in Iran did not have an overall positive attitude toward patients with mental disorders. Although, compared to previous studies, they held a more positive attitude. Positive personal history of psychopharmacological treatment and higher rates of ‘received psychiatric prescriptions per month’ were associated with a more positive attitude. A positive family history of psychiatric inpatient admission was associated with a more negative attitude. As our study faced certain limitations, further research with large sample sizes across different regions of Iran should be conducted to validate the findings of this study.

## Data Availability

The dataset used and analyzed during the current study can be shared by the corresponding author upon reasonable request.
